# Risk of all-cause mortality or hospitalization for pneumonia associated with inhaled β2-agonists in patients with asthma, COPD or asthma-COPD overlap

**DOI:** 10.1186/s12931-022-02295-0

**Published:** 2022-12-20

**Authors:** Joseph Emil Amegadzie, John-Michael Gamble, Jamie Farrell, Zhiwei Gao

**Affiliations:** 1 Faculty of Medicine, Memorial University of Newfoundland, 300 Prince Philip Drive, St. John’s, NL A1B 3V6 Canada; 2 Faculty of Science, School of Pharmacy, University of Waterloo, Waterloo, ON Canada

**Keywords:** Asthma, β2-adrenergic agonists, COPD, Nested case–control

## Abstract

β2-agonists provide necessary bronchodilatory action, are recommended by existing clinical practice guidelines and are widely prescribed for patients with these conditions. We examined the risk of all-cause mortality and hospitalization for pneumonia associated with long-or short-acting β2-agonists (LABA or SABA) or ICS (inhaled corticosteroids)/LABA use. In a nested case–control of 185,407 patients, we found no association between β2-agonist use and the risk of pneumonia in patients with asthma, COPD, or asthma-COPD overlap. In contrast, new SABA [HR 1.82 (95% CI 1.04–3.20)] or LABA [HR 2.77 (95% CI 1.22–6.31)] use was associated with an increased risk of all-cause mortality compared to ICS use in COPD patients.

**Supplementary Information:**

The online version contains supplementary material available at 10.1186/s12931-022-02295-0.

## Introduction

Asthma is a significant public health problem worldwide, causing excess morbidity, mortality, and economic costs [1]. Likewise, chronic obstructive pulmonary disease (COPD) was ranked as the 4th leading cause of death in 2019 and caused considerable morbidity and substantial health care costs [2]. Furthermore, an increasing number of people are affected by asthma-COPD overlap, with 15 to 45% of older adults initially diagnosed with COPD or asthma [3].

β2-agonists provide necessary bronchodilatory action and are recommended by existing clinical practice guidelines, and are widely prescribed for patients with these conditions [4, 5]. Nevertheless, information on the risk of all-cause mortality and pneumonia is limited, and the results are inconsistent [6, 7]. Given the steadily growing trend of β2-agonists-based drug prescriptions (58–185%) in patients with asthma and, more specifically, COPD [8, 9], there is a need to investigate whether these widely prescribed drugs are associated with an increased risk of all-cause mortality and hospitalization for pneumonia.

## Methods

This study was conducted using the United Kingdom Clinical Practice Research Datalink (CPRD) linked to the Hospital Episode Statistics (HES) and Office of National Statistics (ONS) databases, representing the UK’s geographical distribution [10, 11]. The study protocol was approved by the Independent Scientific Advisory Committee of the CPRD (ISAC 18_005RA) and ethical approval was obtained from Health Research Ethics Board at Memorial University, St. John’s, Canada. The study cohort included all males and females diagnosed with asthma, COPD, or asthma-COPD overlap in the CPRD aged 18 or over with a first-ever prescription for a LABA, SABA, combination therapy of ICS/LABA, ICS, LAMA or SAMA.

**Fig. 1:**
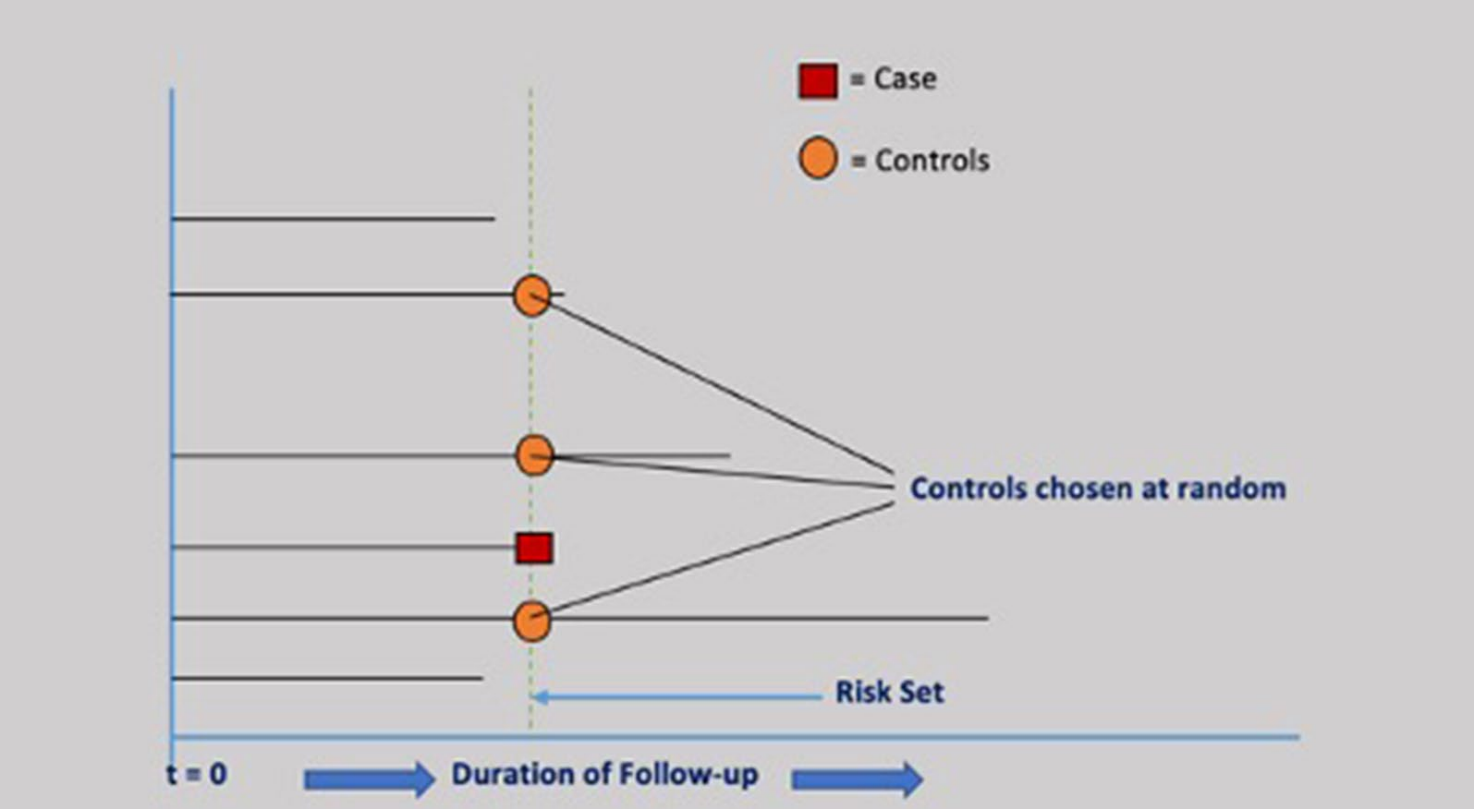
Nested case–control analysis schematic design. 1) Both cases and controls have the same risk of exposure. 2) Matching on follow-up duration ensures that cases and matched controls have the same opportunity time. Cohort entry: at t = 0, i.e., 1^st^ Rx for ICS, ICS/LABA, LABA, LAMA, SABA, or SAMA. Index Date: Date of an event of interest (i.e., Case = Red square) Cohort Exit: A case-patient, emigration, or the end of the study, whichever came first. ICS: inhaled corticosteroid; ICS/LABA: inhaled corticosteroid/long-acting beta-agonist; LABA: long-acting beta-agonist; LAMA: long-acting muscarinic antagonist, SABA: short-acting beta-agonist; SAMA: short-acting muscarinic antagonist

A risk-set sampling method was used to match the case with a random sample from the risk set for each case occurring during the study follow-up. For each case, we randomly selected up to 10 controls within the cohort on the basis of sex, age (± 1 year), date of cohort entry (± 180 days), and duration of follow-up. The case’s index date became the index date for those matched controls selected randomly at the risk-set. The schematic design of the nested case–control analysis employed is shown in Fig. 1.

Details on the study cohort, case–control selection, exposure assessment, covariates, statistical and sensitivity analyses can be found in the Additional file 1.

## Results

**Fig. 2:**
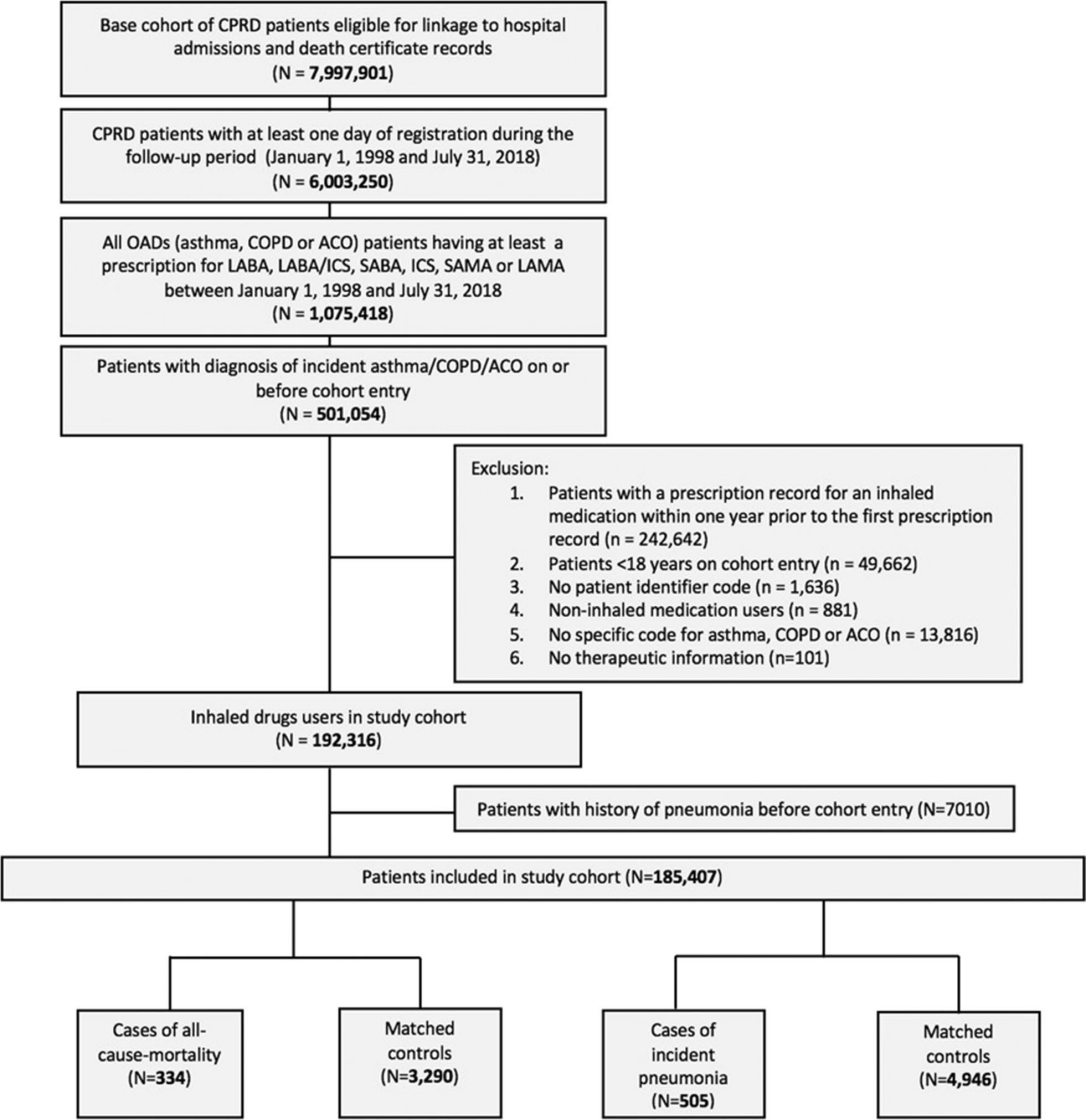
Flowchart of number of patients in the base and study cohort. CPRD: Clinical Practice Research Datalink; OADs: obstructive airway diseases; COPD: chronic obstructive pulmonary disease; ACO: asthma-COPD overlap; ICS: inhaled corticosteroid; LABA: long-acting beta-agonist; LAMA: long-acting muscarinic antagonist; SABA: short-acting beta-agonist; SAMA: short-acting muscarinic antagonist; Rx: prescription

We identified 185 407 eligible patients for the study (Fig. 2), comprising new users of LABA (n = 2,221), SABA (n = 114,600), ICS/LABA combination therapy (n = 5,977), ICS (n = 56,174), LAMA (n = 2,585), and SAMA (n = 3,850).

As per Tables 1 and 2, there were 334 all-cause mortality cases, including 139, 153, and 42 deaths among patients with asthma, COPD, and asthma-COPD overlap, respectively, and 505 new hospitalizations for pneumonia, representing 332, 133, and 40 events among patients with asthma, COPD, and asthma-COPD overlap, respectively. The mean ± SD age at cohort entry with all-cause mortality case-patients was 69.6 ± 14.8, 75.9 ± 9.7 and 75.9 ± 8.0 years for asthma, COPD and asthma-COPD overlap, respectively, and 53.1 ± 19.9, 72.7 ± 9.3 and 72.4 ± 14.3 years for pneumonia case-patients. The baseline characteristics of cases and controls for all-cause mortality (Table 1) and pneumonia (Table 2) are presented.

**Table 1:** Baseline characteristics of all-cause mortality case patients and matched controls

Primary outcome	All-cause mortality					
Types of OADs	Asthma		COPD		Asthma-COPD overlap	
Characteristics	Cases (N = 139)	Controls (N = 1387)	Cases (N = 153)	Controls (N = 1503)	Cases (N = 42)	Controls (N = 400)
Age (years) at inhaled medication initiation	69.6 (± 14.8)	69.1 (± 14.7)	75.9 (± 9.7)	75.2 (± 9.1)	75.9 (± 8.0)	75.4 (± 7.5)
Sex						
Men	75 (54.0)	747 (53.9)	96 (62.8)	941 (62.6)	26 (61.9)	243 (60.8)
Women	64 (46.0)	640 (46.1)	57 (37.3)	562 (37.4)	16 (38.1)	157 (39.3)
Body mass index (kg/m^2^)						
Underweight	9 (6.5)	63 (4.5)	19 (12.4)	169 (11.2)	8 (19.0)	37 (9.2)
Normal	25 (18.0)	384 (27.7)	48 (31.4)	469 (31.2)	6 (14.3)	115 (28.8)
Overweight	39 (28.1)	449 (32.4)	41 (36.8)	406 (27.0)	13 (31.0)	130 (32.5)
Obese	30 (21.6)	284 (20.5)	17 (11.1)	204 (13.6)	15 (35.7)	75 (18.8)
Unknown/missing	36 (25.8)	207 (14.9)	28 (18.3)	255 (17.0)	0 (0.0)	43 (10.7)
Smoking status						
Current	33 (23.7)	184 (13.3)	68 (44.5)	602 (40.1)	17 (40.5)	115 (28.8)
Former	43 (30.9)	437 (31.5)	56 (36.6)	611 (40.7)	18 (42.9)	188 (47.0)
None	50 (36.0)	657 (47.4)	19 (12.4)	192 (12.7)	7 (16.6)	82 (20.5)
Unknown/missing	13 (9.4)	109 (7.8)	10 (6.5)	98 (6.5)	0 (0.0)	15 (3.7)
Alcohol abuse						
None	16 (11.5)	213 (15.4)	19 (12.4)	254 (16.9)	9 (21.4)	69 (17.2)
Former	0 (0.0)	13 (0.9)	5 (3.3)	36 (2.4)	0 (0.0)	9 (2.3)
Current	90 (64.8)	919 (66.3)	97 (63.4)	960 (63.9)	33 (78.6)	272 (68.0)
Unknown/missing	33 (23.7)	242 (17.4)	32 (20.9)	253 (16.8)	0 (0.0)	50 (12.5)
Average systolic blood pressure	141.4 (± 19.1)	140.0 (± 19.8)	137.7 (± 21.7)	141.4 (± 19.5)	140.6 (± 25.6)	141.1 (± 20.2)
Measure of deprivation						
Least deprived	34 (24.5)	327 (23.6)	23 (15.0)	206 (13.7)	5 (11.9)	60 (15.0)
Less deprived	24 (17.3)	325 (23.4)	27 (17.7)	314 (20.9)	7 (16.7)	85 (21.3)
Deprived	33 (23.7)	305 (22.0)	35 (22.9)	308 (20.5)	7 (16.7)	75 (18.8)
More deprived	23 (16.6)	229 (16.5)	32 (20.9)	324 (21.6)	13 (31.0)	101 (25.3)
Most deprived	25 (18.0)	201 (14.5)	36 (23.5)	351 (23.3)	10 (23.8)	79 (19.8)
Unknown/missing	0 (0.0)	0 (0.0)	0 (0.0)	0 (0.0)	0 (0.0)	0 (0.0)
Charlson Index						
0	66 (47.5)	871 (62.8)	71 (46.4)	818 (54.4)	17 (40.5)	209 (52.3)
1	26 (18.7)	221 (15.9)	33 (21.6)	278 (18.5)	8 (19.1)	61 (15.3)
≥ 2	47 (33.8)	295 (21.3)	49 (32.0)	407 (27.1)	17 (40.5)	130 (32.5)
Medications in the year before cohort entry						
ACE inhibitors	52 (37.4)	408 (29.4)	54 (35.3)	506 (33.7)	14 (33.3)	146 (36.5)
Angiotensin receptor blockers	10 (7.2)	73 (5.3)	13 (8.5)	100 (6.7)	0 (0.0)	24 (6.0)
Beta-blockers	20 (14.4)	159 (11.5)	21 (13.7)	204 (13.6)	…^#^	…^#^
Loop diuretics	24 (17.3)	166 (12.0)	41 (26.8)	280 (18.6)	10 (23.8)	81 (20.3)
Thiazide diuretics	35 (25.2)	296 (21.3)	25 (16.3)	314 (20.9)	9 (21.4)	91 (22.8)
Digoxin	7 (5.0)	46 (3.3)	11 (7.2)	77 (5.1)	…^#^	…^#^
Nitrates	17 (12.2)	131 (9.4)	29 (19.0)	186 (12.4)	5 (11.9)	55 (13.8)
Macrolides	22 (15.8)	163 (11.8)	23 (15.0)	177 (11.8)	5 (11.9)	43 (10.8)
Aspirin	42 (30.2)	325 (23.4)	47 (30.7)	449 (29.9)	16 (38.1)	123 (30.8)
Acetaminophen	22 (15.8)	198 (14.3)	46 (30.1)	299 (19.9)	11 (26.2)	65 (16.3)
NSAIDs	29 (20.9)	289 (20.8)	26 (17.0)	246 (16.4)	5 (11.9)	57 (14.3)
Opioids	19 (13.7)	108 (7.8)	22 (14.4)	127 (8.5)	9 (21.4)	36 (9.0)
Insulin	47 (33.8)	451 (32.5)	59 (38.6)	560 (37.3)	18 (42.9)	166 (41.5)
Comorbidities in the year before cohort entry						
Hyperlipidemia	14 (10.1)	123 (8.9)	14 (9.2)	153 (10.2)	6 (14.3)	42 (10.5)
Hypertension	72 (51.8)	581 (41.9)	60 (39.2)	676 (45.0)	18 (42.9)	188 (47.0)
Congenital CVA	…^#^	…^#^	9 (5.9)	52 (3.5)	0 (0.0)	12 (3.0)
Thyroid disease	10 (7.2)	103 (7.4)	9 (5.89)	98 (6.5)	…^#^	…^#^
Liver disease	5 (3.6)	17 (1.2)	0 (0.0)	26 (1.7)	…^#^	…^#^
CHF	5 (3.6)	41 (1.3)	13 (8.5)	79 (5.3)	0 (0.0)	11 (3.1)
Diabetes	20 (14.4)	151 (10.9)	21 (13.7)	158 (10.5)	5 (11.9)	50 (12.5)
Dementia	…^#^	…^#^	…^#^	…^#^	…^#^	…^#^
Renal disease	13 (9.4)	81 (5.84)	12 (7.8)	131 (8.7)	5 (11.9)	44 (11.0)
Atherosclerosis and PVD	17 (12.2)	97 (7.0)	22 (14.4)	152 (10.1)	7 (16.7)	50 (12.5)
Respiratory events and medications in the year before cohort entry						
Physician visits per year						
1–17	47 (33.8)	512 (36.9)	39 (25.5)	419 (27.9)	7 (16.7)	97 (24.3)
18–35	26 (18.7)	386 (27.8)	32 (20.9)	436 (29.0)	8 (19.1)	116 (29.0)
> 36	66 (47.5)	489 (35.3)	82 (53.6)	648 (43.1)	27 (64.3)	187 (46.8)
Moderate or severe exacerbation	28 (20.1)	186 (13.4)	42 (27.5)	293 (19.5)	11 (26.2)	77 (19.3)
Oral corticosteroid	28 (20.1)	185 (13.4)	41 (26.8)	292 (19.4)	11 (26.2)	77 (19.3)
Methylxanthines	…^#^	…^#^	6 (3.9)	33 (2.2)	…^#^	…^#^
Respiratory antibiotics	64 (46.0)	529 (38.1)	83 (54.3)	732 (48.7)	21 (50.0)	173 (43.3)

ICS: inhaled corticosteroid; SABA: short-acting beta2-agonist; LABA: long-acting beta2-agonist; SAMA: short-acting muscarinic antagonist; LAMA: long-acting muscarinic antagonist; LAMA: long-acting muscarinic antagonist; COPD: chronic obstructive pulmonary disease; CI: confidence interval; ACO: asthma-COPD overlap; NSAIDs: non-steroidal anti-inflammatory drugs; CV: cardiovascular; ACE: angiotensin-converting enzyme; CVA: cerebrovascular; CHF: congestive heart failure; PVD: peripheral vascular disease

^**#**^Cells with fewer than 5 events are not shown, per confidentiality policies of the Clinical Practice Research Datalink

**Table 2:** Baseline characteristics of pneumonia cases and matched controls categorized according to OAD diagnoses

Primary outcome	Pneumonia					
Types of OADs	Asthma		COPD		Asthma-COPD overlap	
Characteristics	Cases (N = 332)	Controls (N = 3289)	Cases (N = 133)	Controls (N = 1296)	Cases (N = 40)	Controls (N = 361)
Age (years) at inhaled medication initiation	53.1 (± 19.9)	52.4 (± 19.6)	72.7 (± 9.3)	72.3 (± 9.9)	72.4(± 14.3)	73.2(± 10.5)
Sex						
Men	120 (36.1)	1179 (35.9)	76 (57.1)	754 (58.2)	22 (55.0)	199 (55.1)
Women	212 (63.9)	2110 (64.2)	57 (42.9)	542 (41.8)	18 (45.0)	162 (44.9)
Body mass index (kg/m^2^)						
Underweight	17 (4.7)	179 (3.7)	16 (12.0)	131 (10.1)	7 (17.5)	33 (9.1)
Normal	94 (22.3)	1011 (27.1)	47 (35.3)	383 (29.6)	9 (22.5)	105 (29.1)
Overweight	86 (27.9)	919 (31.8)	28 (21.1)	363 (28.0)	10 (25.0)	120 (33.2)
Obese	87 (30.4)	666 (23.5)	23 (17.3)	257 (19.8)	8 (20.0)	71 (19.7)
Unknown/missing	48 (14.8)	514 (13.9)	19 (14.3)	162 (12.5)	6 (15.0)	32 (8.9)
Smoking status						
Current	87 (16.2)	618 (15.3)	73 (54.9)	551 (42.5)	9 (22.5)	107 (44.8)
Former	76 (30.4)	814 (28.8)	46 (34.6)	556 (42.9)	20 (50.0)	177 (20.4)
None	147 (47.9)	1682 (50.5)	8 (6.0)	138 (10.7)	21 (52.5)	66 (32.2)
Unknown/missing	22 (5.6)	175 (5.5)	6 (4.5)	51 (3.9)	0 (0.0)	11 (2.6)
Alcohol abuse						
None	65 (19.6)	478 (15.7)	32 (24.1)	232 (17.9)	5 (12.5)	57 (15.8)
Former	0 (0.0)	44 (1.8)	5 (3.8)	55 (4.3)	0 (0.0)	13 (3.6)
Current	219 (66.0)	2196 (66.4)	73 (54.9)	839 (64.7)	27 (67.5)	250 (69.3)
Unknown/missing	48 (14.4)	571 (7.6)	23 (17.2)	170 (13.1)	6 (15.0)	41 (11.3)
Average systolic blood pressure	130.0 (± 19.5)	130.5 (± 19.1)	136.2 (± 20.4)	139.2 (± 18.0)	136.9(± 16.5)	137(± 16.8)
Measure of deprivation						
Least deprived	77 (19.8)	799 (24.4)	21 (15.8)	205 (15.8)	5 (12.5)	52 (14.4)
Less deprived	62 (24.5)	776 (23.3)	23 (17.3)	268 (19.8)	13 (32.5)	91 (25.2)
Deprived	69 (21.7	697 (21.6)	21 (15.8)	264 (21.6)	6 (15.0)	74 (20.5)
More deprived	62 (19.2)	559 (16.5)	31 (23.3)	284 (20.4)	6 (15.0)	73 (20.2)
Most deprived	62 (14.8)	453 (14.1)	37 (27.8)	275 (21.9)	10 (25.0)	71 (19.7)
Unknown/missing	0 (0.0)	5 (0.1)	0 (0.0)	0 (0.0)	0 (0.0)	0 (0.0)
Charlson Index						
0	226 (68.1)	2558 (77.8)	61 (45.9)	704 (54.3)	18 (45.0)	200 (55.4)
1	49 (14.8)	309 (9.4)	18 (13.5)	223 (17.2)	12 (30.0)	67 (18.6)
≥ 2	57 (17.2)	422 (12.8)	54 (40.6)	369 (28.5)	10 (25.0)	94 (26.0)
Medications in the year before cohort entry						
ACE inhibitors	67 (20.2)	509 (15.5)	42 (31.6)	451 (34.8)	13 (32.5)	140 (38.8)
Angiotensin receptor blockers	18 (5.4)	119 (3.6)	9 (6.8)	99 (7.6)	6 (15.0)	28 (7.8)
Beta-blockers	26 (7.8)	238 (7.2)	25 (18.8)	203 (15.6)	5 (12.5)	52 (14.4)
Loop diuretics	28 (8.4)	171 (5.2)	35 (26.3)	196 (15.2)	10 (25.0)	60 (16.6)
Thiazide diuretics	46 (13.9)	377 (11.5)	20 (15.0)	247 (19.1)	7 (17.5)	90 (24.9)
Digoxin	7 (2.1)	36 (1.1)	8 (6.0)	47 (3.6)	…^#^	…^#^
Nitrates	18 (5.4)	134 (4.1)	15 (11.3)	143 (11.0)	5 (12.5)	48 (13.3)
Macrolides	65 (19.6)	380 (11.5)	29 (21.8)	206 (15.9)	5 (12.5)	56 (15.5)
Aspirin	55 (16.6)	334 (10.2)	42 (31.6)	358 (27.6)	12 (30.0)	116 (32.1)
Acetaminophen	36 (10.8)	279 (8.5)	29 (21.8)	252 (19.4)	10 (25.0)	58 (16.1)
NSAIDs	62 (18.7)	536 (16.3)	24 (18.1)	194 (15.0)	10 (25.0)	52 (14.4)
Opioids	28 (8.4)	155 (4.7)	22 (16.5)	110 (8.5)	6 (15.0)	42 (11.6)
Insulin	141 (42.5)	1059 (32.2)	62 (46.6)	547 (42.2)	19 (47.5)	158 (43.8)
Comorbidities in the year before cohort entry						
Hyperlipidemia	28 (8.4)	216 (6.6)	16 (12.0)	185 (14.3)	5 (12.5)	55 (11.6)
Hypertension	97(29.2)	816 (24.8)	56 (42.1)	635 (49.0)	18 (45.0)	180 (49.9)
Congenital CVA	6 (1.8)	59 (1.8)	7 (5.3)	49 (3.9)	0 (0.0)	7 (1.9)
Thyroid disease	25 (7.5)	226 (6.9)	6 (4.5)	107 (8.3)	5 (12.5)	36 (10.0)
Liver disease	5 (1.5)	38 (1.2)	…^#^	…^#^	# …	# …
CHF	5(1.5)	41(1.3)	5(3.8)	36(2.8)	# …	# …
Diabetes	31 (9.3)	219 (6.7)	15 (11.3)	144 (11.1)	7 (17.5)	38 (10.5)
Dementia	5 (1.5)	36 (1.1)	…^#^	…^#^	12 (3.3)	5 (1.4)
Renal disease	17 (5.1)	157 (4.8)	17 (12.8)	139 (10.7)	5 (12.5)	37 (10.3)
Atherosclerosis and PVD	14 (4.2)	94 (2.9)	20 (15.0)	143 (11.0)	5 (12.5)	35 (9.7)
Respiratory events and medications in the year before cohort entry						
Physician visits per year						
1–17	98 (29.5)	1403 (42.7)	27 (20.3)	265 (20.5)	5 (12.5)	64 (17.7)
18–35	89 (26.8)	933 (28.4)	30 (22.6)	379 (29.2)	12 (30.0)	108 (29.9)
> 36	145 (43.7)	953 (29.0)	76 (57.1)	652 (50.3)	23 (57.5)	189 (52.4)
Moderate or severe exacerbation	83 (25.0)	372 (11.3)	35 (26.3)	254 (19.6)	11 (27.5)	93 (25.8)
Oral corticosteroid	79 (23.8)	371 (11.3)	35 (26.3)	252 (19.4)	11 (27.5)	92 (25.5)
Methylxanthines	…^#^	…^#^	…^#^	…^#^	…^#^	…^#^
Respiratory antibiotics	193 (58.1)	1250 (38.0)	78 (58.7)	661 (51.0)	22 (55.0)	179 (49.6)

ICS: inhaled corticosteroid; SABA: short-acting beta2-agonist; LABA: long-acting beta2-agonist; SAMA: short-acting muscarinic antagonist; LAMA: long-acting muscarinic antagonist; LAMA: long-acting muscarinic antagonist; COPD: chronic obstructive pulmonary disease; ACO: asthma-COPD overlap; NSAIDs: non-steroidal anti-inflammatory drugs; CV: cardiovascular; ACE: angiotensin-converting enzyme; CVA: cerebrovascular; CHF: congestive heart failure; PVD: peripheral vascular disease

^**#**^Cells with fewer than 5 events are not shown, per confidentiality policies of the Clinical Practice Research Datalink

Among cases with all-cause mortality, controls were less likely to be females than males. On the other hand, Pneumonia patients were more likely to be females with asthma, whereas COPD and asthma-COPD overlap patients were more likely to be males. Except for patients with COPD, case patients were more likely to be obese regarding all-cause mortality. Cases were also more likely to be current smokers, the most deprived, have at least two or more comorbidities (Charlson Index), and prescribed more loop diuretics, aspirin, opioids, and insulin. However, the baseline characteristics of case patients with systolic blood pressure, deprivation (material deprivation), and NSAID prescription were fairly balanced across OADs.

Table 3 shows the multivariate analyses of all-cause mortality and pneumonia after accounting for all baseline variables listed in Tables 1 and 2. Cells with fewer than five events are not permitted to be displayed in the table due to CPRD confidentiality policies.

**Table 3:** Association between use of inhaled β_2_-agonists-based drugs with all-cause-mortality and incidence of hospitalization for pneumonia

OADs/Treatment	All-cause-mortality		Adjusted hazard ratio (95% CI)
	Case patients no. %	Controls no. %	
Asthma 1. ICS (reference) SABA	N = 139 40 (28.8) 90 (64.8)	N = 1387 425 (30.6) 878 (63.3)	1.00 1.11 (0.70–1.76)
COPD 1. ICS (reference) SABA LABA 2. SAMA (reference) SABA ICS/LABA Asthma-COPD Overlap 1. ICS (reference) SABA	N = 153 18 (11.8) 94 (61.4) 12 (7.8) 16 (10.5) 94 (61.4) 13 (8.5) N = 42 9 (21.4) 22 (52.4)	N = 1503 283 (18.8) 845 (56.2) 66 (4.4) 195 (13.0) 845 (56.2) 114 (7.6) N = 400 104 (26.0) 202 (50.5)	1.00 1.82 (1.04–3.20)* 2.77 (1.22–6.31)* 1.00 1.44 (0.82–2.54) 1.45 (0.66–3.19) 1.00 1.10 (0.41–2.96

OADs/Treatment	Pneumonia		Adjusted hazard ratio (95% CI)
	Case patients no. %	Controls no. %	
Asthma 1. ICS (reference) SABA COPD 1. ICS (reference) SABA	N = 332 72 (21.7) 251 (75.6) N = 133 21 (15.8) 84 (63.2)	N = 3289 768 (23.4) 2405 (73.1) N = 1296 193 (14.9) 808 (62.3)	1.00 1.26 (0.93–1.72) 1.00 0.81 (0.45–1.44)
Asthma-COPD Overlap 2. SAMA (reference) SABA	N = 40 5 (12.5) 26 (65.0)	N = 361 35 (9.7) 204 (56.5)	1.00 0.86 (0.27–2.71)

ICS: inhaled corticosteroid; SABA: short-acting β_2_-agonists; LABA: long-acting β_2_-agonists; SAMA: short-acting muscarinic antagonist; LAMA: long-acting muscarinic antagonist; LAMA: long-acting muscarinic antagonist; COPD: chronic obstructive pulmonary disease; CI: confidence interval; ACO: asthma-COPD overlap. * = *p* < 0.05

### β_2_-agonist-based drugs and the risk for all-cause mortality

After controlling for potential confounders, current and new users of SABA (adjusted HR, 1.82 [1.04–3.20]) and LABA (adjusted HR, 2.77 [1.22–6.31]) were significantly associated with an increased risk of all-cause mortality among COPD patients. However, no statistically significant associations were found among asthma or asthma-COPD overlap patients.

### β_2_-agonist-based drugs and hospitalization for pneumonia

As indicated in Table 3, there were no statistically significant associations between the risk of pneumonia and β_2_-agonist-based drugs among patients with asthma, COPD, and asthma-COPD overlap, respectively, after adjusting for potential confounders.

### Sensitivity analyses

Additional file 1: Fig. S2 depicts the results of our sensitivity analyses by using different grace periods for the sub-cohorts of COPD and asthma for the exposure contrast of SABA versus SAMA and the risk of all-cause mortality. The overall results of our sensitivity analyses for all-cause mortality for COPD (top panel) were consistent with those of our primary analyses. Pertaining to asthma patients (bottom panel), the adjusted HR generated in our primary analyses was similar to the one generated in our fixed-effect analysis.

## Discussion

This real-world population-based nested case–control study suggests that among patients with COPD who newly started inhaled β_2_-agonists-based drugs, SABA or LABA monotherapy was associated with a 1.8-fold and 2.8-fold increase in all-cause mortality, respectively, compared with ICS monotherapy. Regarding the risk of pneumonia, our findings indicate that the use of β_2_-agonists-based drugs (SABA) was not associated with an increased risk of pneumonia compared to ICS or SAMA use in patients with asthma, COPD or asthma-COPD overlap. Finally, our findings remained consistent in several sensitivity analyses that explored the overall robustness of our study design and results.

Short-acting β_2_-agonist bronchodilators help relieve COPD symptoms and may be a valuable marker of symptom severity [12]. Using data from 56 primary care and specialty centers in the United States, Dransfield et al. found that a mean SABA use of 3.3 puffs/day was associated with less severe airflow limitation (≥ 50% predicted forced expiratory volume in 1 s [FEV_1_]), compared with 5.2 puffs/day in patients with more severe airflow limitation (< 50% predicted FEV_1_) [13]. It is widely believed that high supplementary SABA use indicates a significant modest risk of exacerbation and hospitalization [14–16]. Our study being novel, is one of the most recent studies to quantify the risk of SABA among COPD patients; we observed that those who started SABA alone are 1.8 times more likely to be associated with all-cause mortality. Thus, the increased use of SABA monotherapy in COPD indicates its ineffectiveness rather than its association with disease severity. Notably, clinical guidelines recommend LAMA or LABA/ICS treatments over regular short-acting β_2_-agonist therapies for patients with exacerbations or persistent breathlessness, also known as patients with moderate or severe COPD [17].

Our observation of a 2.8-fold increased risk of all-cause mortality in COPD patients using LABA monotherapy is consistent with a meta-analysis of RCTs that observed a 2.5-fold increased risk of death in COPD patients using LABA monotherapy compared with placebo [18]. Although this meta-analysis was critiqued for not including the large dataset provided by the 3-year TORCH study, the most significant reductions in death were seen in the combination salmeterol/fluticasone propionate arm rather than the salmeterol monotherapy arm when compared to the placebo. Surprisingly, the sample size obtained after the TORCH study, following a safety call from a 'follow-up assessment,' was entirely inadequate for generating a statistically significant result. The rate of all-cause mortality is regarded as a comprehensive prognostic indicator for any disease; it is dependable and widely regarded as the gold standard in determining the safety of a given therapy [19]. Although it is accepted that there is no cure for COPD, we believe it is time to shift the treatment paradigm for patients with COPD at risk of death from symptomatic relief to long-term treatment improvement. That being so, bronchodilators that alter airway smooth muscle tone are paramount to managing COPD symptoms and exacerbations [20].

Our findings indicate that the use of β_2_-agonist-based drugs is not associated with an increased risk of pneumonia compared with ICS among obstructive airway disease patients with asthma, COPD or asthma-COPD overlap. Even after adjusting for several significant disease severity indicators, including the use of oral corticosteroids, respiratory antibiotics, GP visits, comorbidities, and co-medications, our study still lacks data on lung function tests, such as the FEV_1_ and FEV_1_/FVC ratio, due to significant missing values or its unavailability. Concerning the COPD findings, this must be interpreted with caution due to the low event rates observed in both cases and controls, and differences in clinical presentation and treatment of COPD from country to country. Our findings also provide new evidence on the concerns of potential risk of pneumonia associated with short-acting bronchodilators (SABA, SAMA) among patients with asthma-COPD overlap. This is of particular concern regarding patients with the overlap disease whereby studies of asthma medications have excluded patients with COPD and vice versa.

In conclusion, starting LABA monotherapy or SABA monotherapy treatment was associated with an increased risk of all-cause mortality in patients with COPD. On the other hand, we observed no association between β_2_-agonist-based use and the risk of pneumonia in patients with asthma, COPD or asthma-COPD overlap.

## Supplementary Information

**Additional file 1.** Online Supplemental Material: Methods and Results.

## Data Availability

This study is based on data from the Clinical Practice Research Datalink (CPRD-GOLD) obtained under license from the UK Medicines and Healthcare products Regulatory Agency. Data is available upon official request from CPRD.

## Abbreviations

ICS: Inhaled corticosteroid
SABA: Short-acting β2-agonists
LABA: Long-acting β2-agonists
SAMA: Short-acting muscarinic antagonist
LAMA: Long-acting muscarinic antagonist
LAMA: Long-acting muscarinic antagonist
COPD: Chronic obstructive pulmonary disease
CI: Confidence interval
ACO: Asthma-COPD overlap
TORCH: Towards a Revolution in COPD Health
CPRD: United Kingdom Clinical Practice Research Datalink
GP: General practitioners
BMI: Body mass index
ICD-10: International Classification of Diseases, 10th Revision
ISAC: Independent Scientific Advisory Committee
PPV: Positive predictive value
CVA: Cerebrovascular accident
CHF: Congestive heart failure
PVD: Peripheral vascular disease
ACE: Angiotensin-converting enzyme
HR: Hazard ratio
CI: Confidence interval
NSAIDs: Non-steroidal anti-inflammatory drugs
HES: Hospital episode statistics
ONS: Office of national statistics
