# Supplementary material for: Risk of all-cause mortality or hospitalization for pneumonia associated with inhaled β2-agonists in patients with asthma, COPD or asthma-COPD overlap
Source: Respir Res. 2022 Dec 20;23:364. doi: 10.1186/s12931-022-02295-0 (PMC9764507; doi:10.1186/s12931-022-02295-0)
Supplement: Supplementary file 1 — Additional file 1. Online Supplemental Material: Methods and Results. [file 12931_2022_2295_MOESM1_ESM.docx]

**Additional file: Methods and Results**

**TITLE: Risk of All-cause Mortality or Hospitalization for Pneumonia Associated with Inhaled β2-agonists in Patients with Asthma, COPD or Asthma-COPD Overlap**

Joseph Emil Amegadzie^1^; John-Michael Gamble^2^; Jamie Farrell^1^; Zhiwei Gao^1^

**Affiliations:** ^1^Faculty of Medicine, Memorial University of Newfoundland, St. John’s, Newfoundland, Canada; ^2^Faculty of Science, School of Pharmacy, University of Waterloo, Waterloo, Ontario, Canada.

**Running head:** Inhaled β2-agonists in Patients with Asthma, COPD or Asthma-COPD Overlap

**Corresponding Author**: Dr. Zhiwei Gao, Faculty of Medicine, Memorial University

300 Prince Philip Drive, St. John’s, NL, A1B 3V6 Canada, Email: zhiwei.gao@med.mun.ca

**Additional file: Methods and Results**

**Study Cohort**

The CPRD database includes more than 14 million acceptable (good quality) patients from more than 700 general practitioners (GP) primary care practices.^1,2^ Patients’ data are available for demographics, symptoms and diagnoses, primary care prescriptions (drugs and devices), test results (e.g., spirometry), referrals to a specialist (secondary care), lifestyle information (BMI [body mass index], smoking, alcohol, exercise) hospitalization dates, primary and secondary diagnoses (coded using International Classification of Diseases, 10^th^ Revision [ICD-10]), and related procedures. Patients’ prescriptions data written by GPs are coded into computer records based on the British National Formulary. Data routinely collected in the CPRD is regularly audited and is of high validity (the median proportion of patients with confirmed diagnoses is 89%).^3^

A patient with asthma was defined as having an asthma diagnostic code (readcode) as well as a prescription code (gemscript code) for an asthma medication (including ICS, ICS/LABA, LABA, LAMA, SABA, or SAMA). A validation study found that this patient definition has a positive predictive value (PPV) of 83.3 percent.^4^ COPD was identified as a patient with COPD diagnostic code (read-code) plus a prescription (gemscript code) for COPD medication (including ICS, ICS/LABA, LABA, LAMA, SABA, or SAMA). This definition has a PPV of 87.5% from a COPD validation conducted in the CPRD database.^5^ Also, patients with asthma-COPD overlap were defined as having; 1) asthma readcode and 2) COPD readcode, and 3) an ex/current smoker before the index date.

Cohort entry was defined as the date of first prescriptions during any period from 1-January-1998 to 31-July-2018. We excluded all patients who had taken any inhaler drug within 365 days of their first medication prescription when assessing only new users. Furthermore, to avoid confounding factors associated with severe patients being the first to be prescribed the newest bronchodilator on the market, we only included patients with obstructive airways diseases (OAD) who began LAMA (tiotropium) at least one year after the drug became available in the UK (25-September-2002). Thereby, for LAMA cases and their respective controls, we defined cohort entry as the date of the first prescription on or after September 25, 2003. Patients were followed from the date of study-cohort entry until an event of all-cause mortality or pneumonia occurred, emigration from a CPRD practice site, end of coverage in the database, end of the study period (July 31, 2018), or whichever occurred first.

**Case-Control Selection**

The nested case-control analyses were used to assess the study cohorts described above, in which cases were determined by our primary outcomes of all-cause mortality or pneumonia. This analytical method was adopted due to the time-varying nature of exposure, the size of the cohort, and the long duration of follow-up. Most essentially, the nested case-control analysis is computationally equally efficient, just like the time-dependent survival analysis, while producing identical estimates.^6,7^ To assess only incident cases, patients with an event of hospitalization for pneumonia occurring before the date of cohort entry were excluded. Events definitions were based on International Classification of Disease Version 10 codes (linked HES/ONS data). Thus, the index date was the date of the event for all-cause mortality or admission for pneumonia. The nested case-control analysis was performed separately for all-cause mortality, pneumonia hospitalization, and disease diagnosis (asthma, COPD or asthma-COPD overlap).

For each case that occurred during the study follow-up, a risk-set sampling procedure was employed to match the case with a random sample from the risk set. As a result, each cohort member who was followed and was event-free at the time of the case occurrence was appropriately matched. The adoption of the risk sets allows exposure to be measured at the time of the event of the case, identical to those executed in a Cox proportional-hazards model.

**Exposure assessment**

Cases and controls were classified into 1 of 4 mutually exclusive categories based on their exposure status at the index date;

1. Current and new use of LABA monotherapy was defined by a prescription duration plus a 30-day grace period overlapping the index date;
2. Current and new use of SABA monotherapy was defined by a prescription duration plus a 30-day grace period overlapping the index date;
3. Current and new use of combination therapy ICS/LABA was defined by a prescription duration plus a 30-day grace period overlapping the index date;
4. Not exposed was the reference category for all comparisons. It comprised all patients with ICS, SAMA, or LAMA prescriptions whose duration plus a 30-day grace period overlapped the index date.

The grace period accounted for non-adherence to the prescribed inhaled pharmacotherapy (for example, late make-up for missed doses and/or refills).

The illustration of the assessment of exposure is shown below in **Figure A1.**


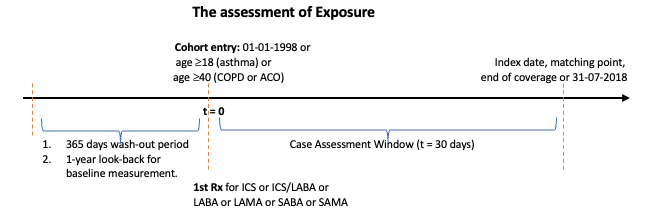


**Figure S1:** The Assessment of Exposure and the comparators

Abbreviations: COPD, chronic obstructive pulmonary disease; ACO, asthma-COPD overlap; ICS, inhaled corticosteroid; SABA, short-acting beta-agonist; LABA, long-acting beta-agonist;

SAMA, short-acting muscarinic antagonist; LAMA, long-acting muscarinic antagonist;

**Covariates**

Covariates included all matching factors (age, sex, and duration of follow-up) used in the study design as well as the following potential confounders measured 365 days before study-cohort entry, which included BMI, smoking status, alcohol abuse, systolic blood pressure, material deprivation, number of physician visits (a measure of health utilization measured before entry), comorbidities (hyperlipidemia, hypertension, congenital CVA (cerebrovascular accident), thyroid disease, liver disease, congestive heart failure [CHF], diabetes, dementia, renal disease, atherosclerosis and peripheral vascular disease[PVD]) Charlson comorbidity index, prescription drugs (macrolides, anti-arrhythmia, ACE (angiotensin-converting enzyme) inhibitors, angiotensin receptor blockers, beta-blockers, loop diuretics, thiazide diuretics, digoxin, aspirin, acetaminophen, opioids, and non-steroidal anti-inflammatory drugs and nitrates).

We also adjusted for the use of respiratory or antibiotics drugs to measure disease severity, which included methylxanthines, oral corticosteroids, respiratory antibiotics and other drugs used (binary; yes/no) in the year before cohort entry, including aspirin, acetaminophen, opioids and non-steroidal anti-inflammatory. We defined moderate or severe exacerbation as a new prescription for prednisolone or hospitalization for asthma, COPD, or asthma-COPD overlap.

**Statistical analysis**

Nested case-control analyses were conducted separately for asthma, COPD, and asthma-COPD overlap. Descriptive statistics were used to summarize the cases' characteristics and matched controls. Categorical variables with more than two categories, type 3 p-values (a p-value indicating the overall effect of all categorical variable levels), were also calculated. We used conditional logistic regression to calculate crude and adjusted hazard ratios (HRs) and 95% confidence intervals for all-cause mortality and hospitalization for pneumonia with new use of LABA, SABA, or ICS/LABA versus ICS, SAMA, or LAMA after controlling for all covariates in our final model. All our models were adjusted for the covariates listed above.

Data analysis was conducted using the IBM cluster's SAS (version 9.4, SAS Institute Inc., Cary, NC).

**Sensitivity Analyses**

We explored a series of sensitivity analyses to examine the robustness of our study design and results. Our study population was restricted to switchers or add-on therapy. For instance, the day patients receive a second inhaler medication; they will begin contributing time at risk to the exposure groups of interest. We repeated the primary analysis by varying the grace period to 0, 45, and 60 days.


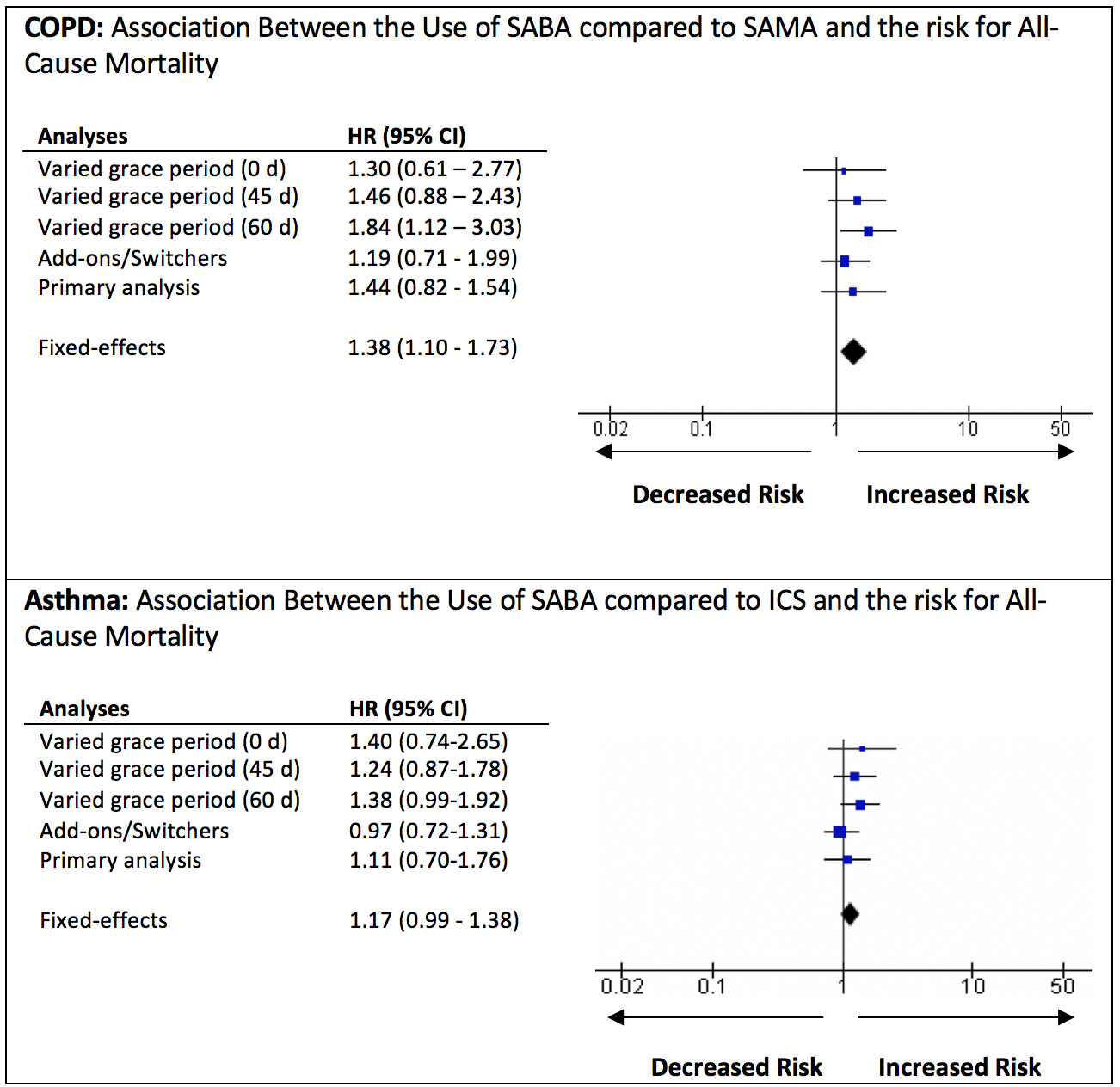


**Figure S2:** Sensitivity Analyses for the Association Between the Use of SABA compared to SAMA or ICS and the risk for All-Cause-Mortality in COPD and Asthma patients, respectively

Abbreviations: COPD, chronic obstructive pulmonary disease; ICS, inhaled corticosteroid;

SABA, short-acting beta-agonist; MACE, major adverse cardiovascular event; HR, hazard ratio

**References**

1. Herrett E, Gallagher AM, Bhaskaran K, et al. Data Resource Profile: Clinical Practice Research Datalink (CPRD). *International journal of epidemiology*. 2015;44(3):827-836. doi:10.1093/ije/dyv098

2. Quint JK, Moore E, Lewis A, et al. Recruitment of patients with Chronic Obstructive Pulmonary Disease (COPD) from the Clinical Practice Research Datalink (CPRD) for research. *NPJ primary care respiratory medicine*. 2018;28(1):21-25. doi:10.1038/s41533-018-0089-3

3. Fournier JP, Azoulay L, Yin H, Montastruc JL, Suissa S. Tramadol Use and the Risk of Hospitalization for Hypoglycemia in Patients With Noncancer Pain. *JAMA internal medicine*. 2014;175(2):186-193. doi:10.1001/jamainternmed.2014.6512

4. Nissen F, Morales DR, Mullerova H, Smeeth L, Douglas IJ, Quint JK. Validation of asthma recording in the Clinical Practice Research Datalink (CPRD). *BMJ open*. 2017;7(8):e017474-e017474. doi:10.1136/bmjopen-2017-017474

5. Quint JK, Müllerova H, DiSantostefano RL, et al. Validation of chronic obstructive pulmonary disease recording in the Clinical Practice Research Datalink (CPRD-GOLD). *BMJ open*. 2014;4(7):e005540-e005540. doi:10.1136/bmjopen-2014-005540

6. Naieni KH, Ostovar A, Danesh A, et al. Comparison of Nested Case-control and Cohort Analysis Methodologies using a District TB Registry Data. *Iranian journal of epidemiology*. 2010;6(2):1-6.

7. Essebag V, Platt RW, Abrahamowicz M, Pilote L. Comparison of nested case-control and survival analysis methodologies for analysis of time-dependent exposure. *BMC medical research methodology*. 2005;5(1):5-5. doi:10.1186/1471-2288-5-5
